# The Impact of Greater Auricular Nerve Injury in Parotidectomy: A Narrative Review of Sensory Outcomes and Quality of Life

**DOI:** 10.3390/jcm15031294

**Published:** 2026-02-06

**Authors:** Heechun Cho, Sang Hoo Park, Younghac Kim, Heejun Yi, Nayeon Choi

**Affiliations:** 1 Department of Otorhinolaryngology-Head and Neck Surgery, Samsung Medical Center, School of Medicine, Sungkyunkwan University, Seoul 06351, Republic of Korea; joysktzxcv@naver.com (H.C.);; 2 Department of Otorhinolaryngology-Head and Neck Surgery, Jeju National University Hospital, College of Medicine, Jeju National University, Jeju 63241, Republic of Korea

**Keywords:** parotidectomy, greater auricular nerve, lobular branch, sensory recovery, quality of life, patient adaptation, surgical morbidity

## Abstract

**Background/Objectives:** Postoperative sensory loss is a frequent morbidity following parotidectomy, yet the necessity of preserving the greater auricular nerve (GAN) during parotidectomy remains debated. While some surgeons prioritize nerve sacrifice for better oncological exposure, others advocate for preservation to maintain quality of life (QoL). This narrative review provides a comprehensive synthesis of current evidence regarding the impact of GAN sacrifice on objective sensory modalities, subjective disturbances, and long-term QoL. **Methods:** A literature search was performed (January 2000–August 2025). Twenty studies, including RCTs and cohorts, were reviewed to synthesize evidence on objective sensory modalities and patient-reported outcomes. Sensory assessments (Semmes–Weinstein monofilaments, VAS, and two-point discrimination) and the POI-8 QoL questionnaire were analyzed. **Results:** GAN preservation, particularly of the lobular branch, is associated with better early sensory recovery (1–6 months). In the long term (>12 months), although the sensory gap narrows between groups, the sacrifice group exhibits significantly higher rates of persistent anesthesia in localized regions, notably the earlobe. Regarding QoL, while global scores often show no significant long-term differences, 35% of patients with GAN sacrifice continue to experience functional limitations in specific activities, such as telephone use or wearing earrings. **Conclusions:** Although patients demonstrate adaptation to sensory loss, GAN preservation offers potential benefits in daily function and comfort; thus, it is advised when oncologically feasible. To overcome the high heterogeneity in current evidence, future multicenter trials utilizing unified objective measurements on predefined regions of interest are necessary to further clarify the functional benefits of nerve preservation and establish definitive surgical guidelines.

## 1. Introduction

Parotidectomy, a common procedure for the treatment of benign and malignant parotid neoplasms, is associated with various morbidities and complications. Among these, the focus has been primarily on preserving the facial nerve and its branches [1,2]. Over time, attention has expanded to include other sources of morbidity. The most frequently reported issue is altered or absent sensation in the area innervated by the greater auricular nerve (GAN) after its sacrifice [2,3].

Anatomically, the GAN emerges from the posterior border of the sternocleidomastoid muscle and ascends toward the inferior pole of the parotid gland [4,5]. It divides into terminal branches, the anterior and posterior branches; notably, the lobular branch—crucial for sensation of the earlobe—often arises from the posterior branch [5,6]. Because of this course, the nerve is continuously encountered and often sacrificed to allow mobilization of the inferior pole of the gland, thus ensuring adequate exposure and oncological safety [1,2,7].

This has given rise to a question: Does routine sacrifice of the GAN, which enables better exposure and manipulation of the parotid gland, outweigh the potential benefit of preserving sensory function through a more conservative approach?

The main reason for preservation is to minimize postoperative sensory morbidity [3]. Early studies reported that preserving the posterior branch of the GAN lessened sensory loss, functional impairment, interference in daily activities (such as shaving or wearing earrings), and the risk of injuries to the affected area [1,2,3,8]. However, some surgeons argue that sacrifice of the GAN allows for a more efficient and potentially safer dissection, particularly for large tumors at the inferior pole [1,2].

Previous studies have utilized heterogeneous endpoints, ranging from objective tools (e.g., Semmes–Weinstein monofilament, two-point discrimination, and touch test) to subjective QoL instruments (e.g., the Parotidectomy Outcome Inventory-8 (POI-8), Visual Analog Scale (VAS)). Furthermore, variations in follow-up duration and anatomical definitions—from single-site testing to detailed regional mapping—complicate direct comparisons. A systematic review by George et al. (2014), which analyzed 13 studies published between 1987 and 2010, established that GAN preservation minimizes sensory disturbance without compromising oncological safety, yet it found conflicting data regarding its impact on overall QoL [2]. However, given the accumulation of recent studies providing long-term data and addressing the significance of the lobular branch, an updated synthesis is warranted.

This narrative review evaluates evidence from 2000 to 2025 to compare the outcomes of GAN preservation versus sacrifice, with a specific focus on these diverse sensory endpoints and QoL and the distinct functional role of the lobular branch.

## 2. Materials and Methods

This study was conducted as a narrative review. A comprehensive search was performed in August 2025 across the PubMed, Medline, Embase, CINAHL, and Cochrane databases using the following terms: “greater auricular nerve”, “nerve sacrifice”, “nerve preservation”, “parotidectomy”, “sensory dysfunction”, “quality of life”, and “morbidity”. References from the relevant articles were also reviewed. We included English-language literature published between 2000 and 2025, encompassing randomized and non-randomized trials, as well as prospective and retrospective case series. Single case reports and “teaching” reviews were excluded. Articles not focusing on specific outcome measures, quality of life, or short- or long-term morbidity were also excluded from the review.

## 3. Results

A total of 1085 articles were identified. After applying advanced filters through an abstract review, 892 articles were excluded. The remaining 193 articles were assessed for eligibility. After final exclusion (single case reports, studies not related, non-relevant articles, unavailable full-text articles, and articles without sufficient data), 22 full-text articles were reviewed. Among them, articles not focusing on specific outcome measures, quality of life, and/or short- or long-term morbidity were also excluded from the review.

Twenty studies were selected for sensory dysfunction analysis, and eight studies were eligible for quality-of-life assessment (Table 1).

The included studies comprised 13 prospective and 7 retrospective studies, including 4 randomized controlled trials and 5 double-blind studies. Two studies were retrospective cohort studies where the GAN was sacrificed in all participants.

Outcome measures varied, with tactile sensitivity being the most common. Methods included subjective scales, the Visual Analog Scale (VAS), sensory index scores, Semmes–Weinstein monofilament testing, and the Touch-Test. Other commonly used outcomes were two-point discrimination, pain sensitivity, temperature sensitivity, sensory impairment, prevalence of sensory symptoms, interference in daily activities, and the global QoL score.

Assessments were typically performed at baseline (pre-operatively) and at various postoperative intervals (1, 6, and 12 months). Most studies followed up until 1 year, while one study only followed up to 6 months [9], and three studies extended the follow-up beyond 2 years [10,11,12]. Additionally, unlike studies with fixed follow-up intervals, five studies reported follow-up periods that varied among patients, with median durations ranging from 22 to 45 months [4,7,8,13,14].

Thirteen studies divided the GAN-innervated areas into regions for detailed testing, ranging from as few as 4 regions to as many as 16. The earlobe was the most consistent site showing differences between the GAN-preserved and GAN-sacrificed groups [1,3,5,6,10,11,12,13,15,16,17,18,19]. However, some studies evaluated only specific areas, such as Suen et al. testing only the earlobe [20], Yokoshima et al. testing only the pinna [9], and Becelli et al. testing the auricle, pinna, and angle of the mandible [21].

**Table 1:** Summary of the studies that dealt with preservation versus sacrifice of the GAN.

Study (Author, Year)	Study Design	GAN Manipulation	Sensory Parameter	Time of Measurement	Exact Value (Preserved vs. Sacrificed/Altered)	*p*-Value
**Biglioli et al., 2002 [5]**	**Prospective Cohort Study**	Posterior Branch Preserved (n = 14) vs. Main Trunk Sacrificed (n = 10) (N = 24)	Tactile Sensitivity (Earlobe)	12 mo	Preserved: 10/14 “Good,” 4/14 “Moderate” Sacrificed: 10/10 “Absent”	NR
			Sharp/Blunt Discrimination (Earlobe)	12 mo	Preserved: 8/14 “Good,” 5/14 “Moderate,” 1/14 “Absent” Sacrificed: 10/10 “Absent”	NR
			Thermal Sensitivity (Heat, Earlobe)	12 mo	Preserved: 5/14 “Good,” 7/14 “Moderate,” 2/14 “Absent” Sacrificed: 10/10 “Absent”	NR
			Thermal Sensitivity (Cold, Earlobe)	12 mo	Preserved: 8/14 “Good,” 6/14 “Moderate” Sacrificed: 10/10 “Absent”	NR
			2 Points Discrimination (Earlobe)	12 mo	Preserved: 5/14 “Good,” 4/14 “Moderate,” 5/14 “Absent” Sacrificed: 10/10 “Absent”	NR
**Vieira et al., 2002 [18]**	**Randomized Prospective Study**	Posterior Branches Preserved (n = 14) vs. GAN Sacrificed (n = 16) (N = 30)	Tactile Sensitivity (Lobule, 0–3 scale)	12 mo	Preserved: Reached pre-op levels (~3.0) Sacrificed: Partial recovery (2.0~2.5)	NR
			Sharp/Blunt Discrimination (Lobule, 0–3 scale)	12 mo	Preserved: Near full recovery (2.5~3.0) Sacrificed: Partial recovery (~1.5)	NR
			2 Points Discriminatino (Lobule, 1–4 scale; 4 worst)	12 mo	Preserved: Poor discrimination (2.0~2.5) Sacrificed: Poor discrimination (1.5)	
**Hui et al., 2003 [12]**	**Prospective Cohort Study**	Posterior Branches Preserved (n = 56) vs. Main Trunk Divided (n = 25) (N = 81)	Tactile sensation	1, 3, 6 mo, 1 yr, 2 yr	Statistically significant difference between groups at all time points in favor of preservation group. Preservation group show near full recovery, while sacrificed group show lasting sensory depression	*p* < 0.05 at all points
			Numbness (subjective scale: none, mild, moderate, severe)	12 mo	**Preserved**: 96.4% “None,” 3.6% “Mild,” 2.8% “Moderate” **Sacrificed**: 13% “None,” 15% “Mild,” 28% “Moderate,” 8% “Severe”	NR
				24 mo	**Preserved**: 100% “None” **Sacrficed**: 15% “None,” 20% “Mild,” 22.5% “Moderate,” 5% “Severe”	NR
**Yokoshima et al., 2004 [9]**	**Prospective Cohort Study**	Posterior Branch Preserved (n = 26) vs. GAN Excised (n = 14) (N = 40)	Tactile sensation (VAS 0–100)	6 mo	Preserved: 66.9 ± 16.2 Sacrificed: 26.6 ± 11.4	*p* = 0.001
**Ryan et al., 2006 [3]**	**Prospective Case Series**	All GAN Sacrificed (n = 22)	Prevalence of Anesthesia	12 mo	50% of patients had anesthesia, with average 18% of area innervated by GAN	NR
			Prevalence of Paresthesia	12 mo	86% of patients had paresthesia, with average 48% of area innervated by GAN	NR
**Min et al., 2007 [15]**	**Prospective Cohort Study**	Posterior Branch Preserved (n = 24) vs. Sacrificed (n = 22) (N = 46)	Tactile sensation (Sensory Index Score)	1 week	Preserved: 41.87 ± 20.62 Sacrificed: 62.11 ± 14.06	*p* = 0.015
				1 month	Preserved: 24.91 ± 12.19 Sacrificed: 46.11 ± 14.95	*p* = 0.010
				12 mo	Preserved: 9.29 ± 4.68 Sacrificed: 7.85 ± 5.61	*p* = 0.485
**Suen et al., 2007 [20]**	**Prospective Cohort Study**	GAN Preserved (n = 10) vs. Sacrificed (n = 11) (N = 21)	Light Touch (Earlobe, minimum diameter that could be felt with an anesthesiomete	12 mo	**Preserved**: 3.5400 mm **Sacrificed**: 2.9860 mm	*p* = 0.026
			2 Points discrimination (Earlobe, minimum diameter)	12 mo	**Preserved**: 16.9 mm **Sacrificed**: 18.4 mm	*p* = 0.267
			Sharp pain (Earlobe, % of patient)	12 mo	**Preserved**: 27.3% **Sacrificed**: 20.0%	*p* = 0.016
			Perceived numbness (Earlobe, % of patient)	33–66 mo	**Preserved**: 67% **Sacrificed**: 28%	*p* > 0.05
**Ryan et al., 2009 [16]**	**Prospective Case Series**	All GAN Sacrificed (n = 19)	Prevalence of Anesthesia	4–5 years	47% of patients had anesthesia, with average 12% of region innervated by GAN	NR
			Prevalence of Paresthesia	4–5 years	58% of patients had paresthesia, with average 27% of region innervated by GAN	NR
**Hu et al., 2010 [1]**	**Randomized Controlled Trial**	Post. branch preserved, Lobular branch sacrificed (n = 29) vs. Post. Branch sacrificed, Lobular branch preserved (n = 33) vs. Total sacrificed (n = 13) (N = 75)	Tactile Sensitivity (Lobule, % recovering pre-op level)	12 mo	Lobular: 96.4% Post. Preserved: 63.0% Sacrificed: 57.1%	*p* < 0.05
			Pain Sensitivity (Lobule, % recovering pre-op level)	12 mo	Lobular: 100% Post. Preserved: 40.7% Sacrificed: 21.4%	*p* < 0.05
**Yang et al., 2011 [10]**	**Prospective Cohort Study**	Preserved (n = 15) vs. Sacrificed (n = 14) (N = 29)	Light Touch (Lobule)	12 mo	Significant difference favoring preservation.	*p* = 0.04
			Temperature (Infra-auricular)	12 mo	Significant difference favoring preservation.	*p* = 0.03
				45 mo	Significant difference favoring preservation.	*p* = 0.001
			No other significant differences shown in other time period (1, 3, 6, 12, 45 mo), area, sensation (2 points discrimination, pain sensation)			NR
**Becelli et al., 2014 [21]**	**Retrospective Cohort Study**	GAN Spared (n = 76) vs. Sacrificed (n = 2)	Subjective Sensitivity	12 mo	Spared: All patients had good sensation. Sacrificed: Both patients reported numbness.	NR
**Grammatica et al., 2015 [13]**	**Retrospective Cohort Study**	Posterior Branch Preserved (n = 42) vs. Sacrificed (n = 13) (N = 55)	Tactile Sensitivity (Lobule, % normal)	Median 24 mo (12–46 mo)	Preserved: 55% Sacrificed: 31%	*p* = 0.04
			Tactile Sensitivity (Other areas, % normal)	Median 24 mo (12–46 mo)	Preserved: 16.7–66.7%(depending on area) Sacrificed: 0–61.5% (depending on area)	NR
			Heat Sensitivity (% normal)	Median 24 mo (12–46 mo)	Preserved: 11.9–73.8% (depending on area) Sacrificed: 0–53.8% (depending on area)	NR
			Cold Sensitivity (% normal)	Median 24 mo (12–46 mo)	Preserved: 21.4–81.0% (depending on area) Sacrificed: 7.7–76.9% (depending on area)	NR
**Moretti et al., 2015 [6]**	**Prospective Cohort Study**	Post. Branch preserved, Lobular branch sacrificed (A/n = 20) vs. Post. Branch and Lobular branch preserved (B/n = 20) vs. Total sacrificed (C/n = 20)	Tactile Sensitivity (Lobule, VAS 0–100)	12 mo	Post. Branch and Lobular Branch Preserved: 90.0 ± 4.2 Post. Branch Preserved, Lobular Branch Sacrificed: 51.2 ± 6.8 Total Sacrificed: 45.2 ± 7.6	B vs. A, B vs. C significant
			Tactile Sensitivity (Pre-auricular region, VAS 0–100)	12 mo	Post. Branch and Lobular Branch Preserved: 97.2 ± 2.5 Post. Branch Preserved, Lobular Branch Sacrificed: 94.2 ± 4.6 Total Sacrificed: 92.2 ± 4.7	B vs. C significant
			Tactile Sensitivity (Sup.-auricular region, VAS 0–100)	12 mo	Post. Branch and Lobular Branch Preserved: 95.0 ± 3.2 Post. Branch Preserved, Lobular Branch Sacrificed: 96.7 ± 2.9 Total Sacrificed: 90.5 ± 3.9	A vs. C, B vs. C significant
			Tactile Sensitivity (Post.-auricular region, VAS 0–100)	12 mo	Post. Branch and Lobular Branch Preserved: 92.7 ± 3.0 Post. Branch Preserved, Lobular Branch Sacrificed: 95.0 ± 3.9 Total Sacrificed: 57.0 ± 4.7	A vs. C, B vs. C significant
			Tactile Sensitivity (Infra-auricular region, VAS 0–100)	12 mo	Post. Branch and Lobular Branch Preserved: 90.7 ± 4.0 Post. Branch Preserved, Lobular Branch Sacrificed: 87.7 ± 4.1 Total Sacrificed: 69.,7 ± 4.7	A vs. C, B vs. C significant
**Grosheva et al., 2017 [11]**	**Prospective Cohort Study, Multicenter**	GAN Preserved (n = 93) vs. Ligated (n = 33) (N = 126)	Tactile Sensation (Lobule, % Positive in Touch Test)	12 mo	Preserved: 59% Ligated: 24%	*p* = 0.013
				24 mo	Preserved: 70% Ligated: 31%	*p* = 0.019
			Tactile Sensation (Antitragus, % Positive in Touch Test)	24 mo	Preserved: 71% Ligated: 31%	*p* = 0.045
			Subjective Sensation loss (POI-8 Score item, % of patients)	12 mo	Preserved: 83% Ligated: 88%	*p* > 0.05
			\			
**Lee et al., 2017 [17]**	**Retrospective Cohort Study**	Preserved (n = 39) vs. Sacrificed/Injured (n = 13) (N = 52)	Light Touch (Lobule, g/mm^2^, mean minimum pressure thresholds change compared to pre-op level)	12 mo	Preserved: 1.34 g/mm^2^ Sacrificed/Injured: 1.18 g/mm^2^	*p* = 0.168
			No significant differences in other area (superior helix, tragus, pre-, infra-, post-auricular area)			
**Bulut et al., 2019 [14]**	**Retrospective Cross-sectional Study**	GAN Preserved (n = 29) vs. Sacrificed (n = 108) (N = 137)	Sensory Impairment (POI-8 Score item)	2 weeks	Preserved: 2.1 Sacrificed: 2.8	*p* = 0.017
				>5 yr (mean 100 mo)	Preserved: 1.3 Sacrificed: 1.7	*p* = 0.145
**Yan et al., 2021 [4]**	**Retrospective Cohort Study**	GAN Preserved (n = 30) vs. Sacrificed (n = 37) (N = 67)	Sensory Symptoms (Lobule, Palet et al. 8-item survey, % of patient)	Mean 45 mo	Preserved: 40.0% Sacrificed: 64.9%	*p* = 0.042
			Sensory Symptoms (Concha, Palet et al. 8-item survey, % of patient)	Mean 45 mo	Preserved: 13.3% Sacrificed: 35.1%	*p* = 0.041
			Pain (POI-8 Score item)	Mean 45 mo	Preserved: 0.87 Sacrificed: 1.05	*p* = 0.591
			Sensory Impairment (POI-8 Score item)	Mean 45 mo	Preserved: 0.97 Sacrificed: 1.19	*p* = 0.456
			No significant differences between other areas, symptom types-frequency-duration			
**Al-Aroomi et al., 2022 [19]**	**Prospective Cohort Study**	GAN Preserved (n = 28) vs. Sacrificed (n = 22) (N = 50)	Tactile Sensitivity (Mandibular Body, % Numb)	1 mo	Preserved: 46.4% Sacrificed: 90.9%	*p* = 0.001
			Tactile Sensitivity (Lobule, % Numb)	1 mo	Preserved: 39.3% Sacrificed: 72.7%	*p* = 0.02
			Tactile Sensitivity (Mandibular Body, % Numb)	9 mo	Preserved: 21.4% Sacrificed: 54.5%	*p* = 0.017
			Tactile Sensitivity (Any numbness, % Numb)	12 mo	Preserved: 21.4% Sacrificed: 40.9%	*p* = 0.14
			Sensory Impairment (POI-8 Score item)	12 mo	Preserved: 0.39 Sacrificed: 0.86	*p* = 0.039
**Patel et al., 2001 [8]**	**Retrospective Cross-sectional survey**	All GAN Sacrificed (n = 53)	Symptom Prevalence (% of patients)	Median 22 mo (3–69 mo)	0.57	NA
			Mean Number of Symptoms	2.3 (0–11 mo), 1.7 (12–23 mo), 0.5 (24–35 mo), 0.7 (36–47 mo), 0.3 (48–59 mo), 0.2 (60–71 mo)		*p* < 0.001
**Galli et al., 2015 [7]**	**Retrospective Case Series**	All GAN Sacrificed (n = 191)	Symptom Prevalence (% of patients)	Median 34 mo (5–81 mo)	0.717	NA
			Mean Number of Symptoms	2.3 (0–11 mo), 1.8 (12–23 mo), 1.8 (24–35 mo), 1.8 (36–47 mo), 1.7 (48–59 mo), 1.1 (60–71 mo), 1.0 (72–84 mo)		*p* < 0.001

NR: not reported.

## 4. Discussion

The significant heterogeneity in outcome measurements, length of follow-up, blinding status, tested anatomical sites, and the inclusion of uncontrolled studies precluded a high-quality meta-analysis. Therefore, a comprehensive narrative review paper was written. Findings of this study should be interpreted as associative rather than strictly causal, due to the inherent limitations of the available literature.

### 4.1. Objective Sensory Outcomes: A Synthesis of Modality-Specific Evidence

Heterogeneity in assessment tools for objective sensory outcomes—ranging from quantitative aesthesiometry to qualitative bedside tests—has two critical implications. First, it precludes a quantitative meta-analysis. Second, the use of less-sensitive qualitative tools in some studies may fail to detect subtle deficits, potentially leading to an underestimation of the benefits of nerve preservation. Consequently, while the synthesized data generally suggest a trend favoring GAN preservation over sacrifice, these findings must be interpreted with caution, taking into account the precision and heterogeneity of measurement methods employed [2,12,18].

Tactile sensation, referred to as “light touch” in some studies [10,17,20], was the most frequently studied outcome, reported in 13 out of 19 studies. Despite differences in measurement methods, preservation groups tended to exhibit superior outcomes [2]. Six studies utilizing Semmes–Weinstein monofilaments reported better outcomes with preservation, while one study found no significant differences observed [3,11,12,13,17,20]. For instance, Hui et al. reported lower pressure thresholds in the preservation group up to two years post-surgery [12]. Similarly, two studies employing VAS to quantify tactile sensation concluded that preserved groups exhibit significantly better tactile sensitivity, particularly in the ear lobule and infra-auricular region [6,9]. Even simpler methods, such as light touch testing with cotton wool or a brush, have demonstrated a significant benefit associated with preservation in three studies [1,10,18].

Beyond tactile sensitivity, seven studies investigated other modalities, including pain (or sharp-blunt discrimination) [1,5,10,15,18,20], tactile discrimination (two-point discrimination) [5,10,18,20], and thermal sensitivity [5,10,13,18]. Findings in these studies imply that preservation may help maintain a broad spectrum of sensory functions, including those mediated by A-delta and C fibers (pain and temperature) and A-beta fibers (fine discrimination) [1,5]. Comprehensive assessment by Biglioli et al. and Vieira et al. showed that preservation groups achieved “Good” or “Moderate” or near preoperative levels across these modalities by 12 months, contrasting with the “Absent” or partial, stabilized recovery observed in the sacrificed group [5,18].

However, results are not uniform. Suen et al. reported improved pain sensitivity with preservation, yet no significant difference was found in two-point discrimination [20]. Yang et al. observed no significant long-term difference in above modalities in most region, with exception of infra-auricular area [10].

### 4.2. Sensory Recovery in Short-Term vs. Long-Term Results

The difference in the degree of sensory recovery between the preserved and sacrificed groups appears most evident in the early postoperative period. However, this difference tends to evolve over time, leading to more complex long-term results [2].

#### 4.2.1. Short-Term Results (First 1–3 Months)

Most prospective and RCT data indicate an association between GAN preservation and reduced initial sensory deficit. Min et al. defined a “sensory index score” and found significantly better results in the preserved group at both 1 week (41.87 ± 20.62 vs. 62.11 ± 14.06, *p* = 0.015) and 1 month (24.91 ± 12.19 vs. 46.11 ± 14.95, *p* = 0.010) after surgery, showing a near 50% reduction in the initial sensory deficit with preservation [15]. Hui et al. also reported a statistically significant difference in favor of preservation at 1 and 3 months (*p* < 0.05) [12]. Al-Aroomi et al. demonstrated significantly better results in tactile sensitivity in the region of the mandibular body (46.4% vs. 90.9%, *p* = 0.001) and lobule (39.3% vs. 72.7%, *p* = 0.02) at 1 month after surgery; however, these differences were not seen after 6 months and 1 year [19]. Yokoshima et al. found significantly higher VAS scores for tactile sensation in the preserved group at 2, 3, and 6 months after surgery (*p* < 0.008) [9]. Other studies demonstrated a similar trend, showing that the GAN preserved group maintained better sensory function early in the postoperative period [14,18,20,21]. Collectively, these studies suggest that preserving the GAN provides a potential benefit by reducing the severity of the initial sensory deterioration.

In contrast, three studies reported that preserving the GAN was not associated with significant benefit in the short-term [10,11,17]. Lee et al. measured the change in tactile pressure threshold from preoperative levels and found no significant difference between the preserved and sacrificed groups at 1, 3, 6, or 12 months in most tested areas [17]. Yang et al. reported no significant difference between the two groups in light touch after 1 week, 1 month, and 3 months [10]. Grosheva et al., in a large multicenter trial, tested 16 anatomical areas and found no significant difference in tactile sensation between the two groups at 6 months, while the preserved group showed better result at 12 and 24 months [11].

The discrepancy between these two opposing results likely stems from differences in outcome definitions and measurement protocols rather than simple errors. First, the studies lacking short-term benefit often employed methods that could mask localized effects. [11,17] For example, the testing of 16 anatomical points by Grosheva et al. sums up all the results from each of the 16 points. While this approach robustly assesses the wider facial region, it may dilute site-specific deficits [11]. Since the GAN primarily innervates the earlobe and angle of the mandible, averaging these scores with unaffected peripheral areas could mask statistically significant differences. Secondly, Lee et al. analyzed the degree of threshold change relative to preoperative baseline, rather than comparing absolute postoperative thresholds between groups. Their finding that both groups experienced a similar relative decline in sensation suggests that surgical elevation of the skin flap affects sensation regardless of nerve status [17]. However, this valid observation does not contradict other studies showing that the absolute sensory threshold remains better in preserved groups. Lastly, Yang et al. utilized a qualitative light touch test using cotton wisps, which lacks the sensitivity of quantitative aesthesiometry required to detect subtle threshold changes [10]. Therefore, these conflicting studies highlight that while global or relative sensation might not show overt difference, especially when measured with less-sensitive tools, absolute sensory function in specific target areas (e.g., lobule) is consistently better preserved when the GAN is preserved.

#### 4.2.2. Long-Term Results (>12 Months)

The results regarding long-term sensory recovery were more complicated, with some studies suggesting a persistent effect, while others showed a lack of significant difference as time passes.

Multiple high-quality randomized trials and prospective studies suggest that certain areas, particularly the earlobe, may benefit from GAN preservation for the restoration of sensation, whereas other areas previously innervated by the GAN demonstrate general spontaneous recovery regardless of nerve manipulation [2,5,11,18]. Grosheva et al. found, in a prospective multicenter trial, that at 12 months, a higher percentage of patients in the preserved group recovered tactile sensation in the earlobe (59% vs. 24%, *p* = 0.013). The difference persisted until 24 months after the surgery (70% vs. 31%, *p* = 0.019), and also extended to the antitragus (71% vs. 31%, *p* = 0.045) [11]. Grammatica et al. also found the preserved group showed better tactile sensitivity in the lobule at a median follow-up of 24 months (55% vs. 31%, *p* = 0.04) [13]. Vieira et al., Yang et al., and Hui et al. similarly reported persistent differences favoring the preserved group at 12 months and after [10,12,18]. These results were also seen in other modalities. Suen et al. showed a higher percentage of patients showing normal pain sensation in the preserved group at 12 months in the earlobe (27.3% vs. 20.0%, *p* = 0.016) [20]. Biglioli et al. reported that at 12 months, 100% of patients in the sacrificed group had “absent” tactile sensitivity, sharp/blunt discrimination, thermal sensitivity, and tactile discrimination (two-point), while most patients in the preserved group showed “good” or “moderate” sensation [5].

Two studies also highlight the potential importance of preserving certain anatomical structures, namely the lobular branch, for the normal sensory recovery of the ear lobe. Hu et al. reported that at 12 months, recovery of tactile sensitivity in the lobule was 96.4% in the lobular branch preserved group, 63.0% in the posterior branch preserved only group, and 57.1% in the total GAN sacrificed group (*p* < 0.05) [1]. A similar study by Moretti et al. found that VAS scores for tactile sensitivity in the lobule were highest in the group where both the posterior and lobular branches were preserved, compared with groups where only the posterior branch was preserved or the total GAN was sacrificed (90.0 vs. 51.2 vs. 45.2, *p* < 0.05) [6].

However, four studies report that the statistically significant gap between the groups’ sensory recovery narrows or closes by the 12-month mark [14,15,17,19]. Min et al. found no significant difference in their sensory index score at 12 months (9.29 ± 4.68 vs. 7.85 ± 5.61, *p* = 0.485) while showing a difference in short-term [15]. Al-Aroomi et al. also demonstrated no significant difference in the number of patients reporting numbness at 12 months (21.4% preserved vs. 40.9% sacrificed, *p* = 0.140), although the recovery rate was higher in the preserved group (78.5% vs. 59.1%) [19]. Similarly, long-term follow-up by Bulut et al. using the POI-8 sensory item, found no significant difference at more than 5 years (1.3 preserved vs. 1.7 sacrificed, *p* = 0.145) [14]. Lee et al. showed no difference between the two groups in threshold change compared to preoperative level (*p* = 0.168) [17].

The convergence observed in these studies can be attributed to several factors. Studies such as Min et al. used a comprehensive measure, the “sensory index score” in this case, which could diminish differences observed in certain key areas, such as the earlobe, by averaging with other areas that showed no significant difference [15]. Heterogeneity of outcome measurement could also explain the phenomenon, as Lee et al. compared the degree of change from preoperative levels, rather than comparing the absolute sensory function at a certain time period [17]. Another factor explaining this convergence, as suggested by some studies, is the potential for long-term neural regeneration and collateral innervation from adjacent nerves (e.g., lesser occipital, auriculotemporal, transverse cervical) [1,15,19]. These mechanisms may explain why differences measured by broad or comprehensive measurement between groups diminish over time, even if localized deficits in certain areas—e.g., the earlobe—still persist [2].

In summary, sensory recovery occurs in all patients over the long term, and the extent of the difference between the GAN preserved and GAN sacrificed groups becomes less distinct. However, studies focusing on specific areas suggest that GAN preservation is associated with better long-term sensory recovery in key regions, such as the earlobe [1,6,11].

### 4.3. Subjective Sensory Disturbance

Apart from objective testing, subjective sensory disturbance was investigated in five studies [3,8,12,16,21].

Ryan et al. reported in a cohort of patients with sacrificed GAN that at 1 year after surgery, 50% of patients still had areas of anesthesia, covering an average of 18% of the GAN-innervated territory; this decreased to 47% of patients with an average area of 12% at 4–5 years [3,16]. Similarly, at 1 year, 86% of patients had paresthesia involving an average area of 48%, which declined to 47% of patients with an average area of 27% at 4–5 years [3,16]. This demonstrates that complete anesthesia can be permanent after GAN sacrifice. It also suggests a process where the initial anesthesia area is replaced by paresthesia as nerve regeneration occurs [3,16].

In contrast, the preserved group showed better subjective outcomes. Hui et al. reported at 24 months, 100% of patients in the GAN preserved group reported “no numbness” in surveys, compared to only 13% in the sacrificed group [12]. Similarly, Becelli et al. showed that while two patients in the GAN-sacrificed group reported numbness after 12 months, all 76 patients in the preserved group reported good subjective sensation [21]. This difference suggests that preservation is associated with a reduction in persistent subjective numbness.

### 4.4. Impact on Quality of Life

Ten out of the 20 studies assessed the impact of GAN manipulation on postoperative quality of life (QoL). Various measurements were employed, including VAS, questionnaires (commonly used are Parotidectomy Outcome Inventory-8 (POI-8) scores, a validated questionnaire used in Patel et al. [8]). The most common outcome measures used were the overall score of QoL (used specifically in POI-8), interference with daily activities, the presence of abnormal symptoms, sensory impairment, and perceived numbness.

The POI-8 questionnaire, developed by Baumann et al., is the most widely used validated patient-reported outcome measure (PROM) in this field, specifically designed to assess patient-reported QoL following parotid surgery. It evaluates possible symptoms using a six-point scale ranging from 0 (“no problem”) to 5 (“problem cannot be worse”) [22]. The questionnaire consists of eight distinct items addressing postoperative sequelae, such as Frey’s syndrome, facial nerve palsy, pain, and sensory impairment. The total score, ranging from 0 to 40, represents a summary of the patient’s overall postoperative state, with lower scores indicating fewer symptoms and better QoL.

Among the four studies utilizing the POI-8, two compared total scores and found no significant difference between the GAN sacrifice and preservation groups [4,14]. This lack of difference is likely attributable to the “dilution effect” inherent in the POI-8 structure, where six of the eight items assess non-sensory outcomes. If other surgical outcomes are satisfactory, a patient may achieve a low global POI-8 score (indicating better QoL) and report a general sense of well-being despite a significant sensory deficit, which is reflected by a high score on Item 2 [4,14].

However, relying solely on the total score obscures the specific impact of nerve sacrifice. When determining the effect of GAN preservation, it is crucial to analyze item 2 (“sensory impairment in the area of surgery and/or neck”) independently. Studies isolating this specific item consistently showed significant deficits in the sacrifice groups, findings that are often diluted when comparing only the total score [4,14,19]. For instance, Al-Aroomi et al. reported a significant difference in the “sensory impairment” of the POI-8 survey at 12 months, favoring the preserved group (0.39 vs. 0.86, *p* = 0.039), while no significant differences were observed in the other seven items [19]. Similar findings are summarized in Table 2.

Apart from POI-8, three studies used QoL assessment based on a questionnaire utilized by Patel et al., focusing more specifically on sensory aspects of postoperative sequelae [4,8,13,15]. These findings consistently show that although sensory deficits persist during follow-up, their impact on overall QoL remains minimal. Min et al. found that, regardless of GAN manipulation, the majority of patients reported “almost no interference” with daily activities, with no significant differences in QoL between the preserved and sacrificed groups [15]. Grammatica et al. reported that only the size of the hypoesthetic area was significantly larger in the sacrificed group, while all other survey items—including frequency, degree of bother, and interference with daily life—showed no significant differences [13]. Yan et al. also reached a similar conclusion; while the sacrificed group showed higher frequency of symptoms in certain key areas (such as the ear lobule or concha), there were no significant differences in interference with daily activities or in the results of other QoL items [4].

Consequently, while preservation appears to be associated with better objective and subjective sensory scores in specific regions, these specific benefits do not consistently translate to a significant difference in overall function and global QoL scores.

Large cohort studies also report similar results: patients with a sacrificed GAN show a high prevalence of symptoms, but a low impact on QoL [7,8]. Galli et al., at a median follow-up of 34 months, reported that more than 70% of patients experienced abnormal sensations; however, the degree of bother was generally mild and the discomfort did not significantly affect daily activities [7]. Similarly, Patel et al. found that although 57% of patients reported at least one abnormal symptom, 90% of those symptomatic patients reported “no interference or almost none with their daily activities” [8]. The number and degree of bothersome symptoms decrease over the years, suggesting patient adaptation to the sensory sequelae [7,8,16]. Although direct measurement of this adaptation was not performed in the reviewed studies, the longitudinal trend of diminishing subjective scores in the presence of stable objective deficits suggests that patients adapt to the altered sensation over time.

The discrepancy between overall QoL and specific functional morbidity is best illustrated in the long-term cohort study by Ryan et al. [3,16]. In patients with GAN sacrifice, the prevalence of functional deficit was 91% at 1 year postoperatively, decreasing to 35% at 4–5 years. Commonly reported limitations included telephone usage, wearing earrings, shaving and combing hair. While the prevalence of these limitations decreased over time, they persisted even at 4–5 years (respectively 40%, 25%, 13%, 10%). During that same period, however, 90% of patients rated the interference of daily living as “none” or “almost none”, with the remaining 10% reporting it as “mild” [3,16]. This stark contrast confirms that global QoL instruments often fail to capture specific sensory morbidities, due to patients adapting to task-specific limitations and not perceiving these hindrances as critical to their overall well-being.

The collective evidence from the reviewed literature can be summarized as follows:

- **Objective outcomes**: GAN preservation is associated with improved multiple sensory modalities, particularly in the earlobe and lobule.
- **Short-term recovery**: Preservation appears to reduce immediate postoperative sensory loss.
- **Long-term recovery**: Differences between groups narrow over time, but preservation—especially of the lobular branch—may provide sustained benefit in key regions.
- **Subjective symptoms**: Preservation is associated with lower prevalence of numbness and paresthesia, while sacrifice may lead to permanent anesthesia in some patients.
- **Quality of life**: Despite measurable sensory deficits, global QoL appears largely unaffected due to patient adaptation.

## 5. Conclusions

This narrative review suggests that preserving the greater auricular nerve (GAN), particularly the lobular branch, is associated with minimized sensory morbidity after parotidectomy and supports better functional recovery, most notably in the early postoperative period [5,11,12,15,18]. While the advantage of preservation in sensory recovery may diminish over the long term, preservation may still offer sustained, site-specific benefits, particularly in the earlobe. Although overall QoL often appears uncompromised due to measurement instrument limitations and patient adaptation, specific functional impairments persist. Therefore, consideration for GAN preservation is advised when oncologically feasible, because it may offer potential benefits in terms of daily function and patient comfort [4,7,8,14,15].

Current literature is limited by a reliance on single-center studies with heterogeneous outcome measurements, varying in both assessment tools and tested areas. These limitations prevent a quantitative comparison and the establishment of clear surgical guidelines. Moreover, the majority of existing studies are confined to follow-up periods of less than two years, leaving the long-term trajectory of sensory changes unexplored.

Consequently, future research should prioritize well-designed, multicenter studies that employ unified, objective sensory testing tools on predefined anatomical regions of interest, specifically the lobule. Crucially, these studies must incorporate significantly extended follow-up periods to accurately track the later stage of sensory recovery and adaptation.

## Figures and Tables

**Table 2:** POI-8 Sensory Impairment scores in studies.

Study (Author, Year)	Follow-Up Time	Preserved Group Score (Mean)	Sacrificed Group Score (Mean)	*p*-Value
Bulut et al., 2019 [14]	2 weeks	2.1	2.8	0.017
	>5 yr	1.3	1.7	0.145
Grosheva et al., 2017 [11] *	12 mo	83% reported loss	88% reported loss	>0.05
Yan et al., 2014 [4]	Mean 45 mo	0.97	1.19	0.456
Al-Aroomi et al., 2021 [19]	12 mo	0.39	0.86	0.039

Note: POI-8 scores are reported on a 0–5 scale, where lower values indicate less impairment. * Grosheva et al. reported the percentage of patients with subjective sensation loss rather than mean POI-8 scores, and therefore results are not directly comparable with mean values from other studies [11].

## Data Availability

No new data was created or analyzed in this study.
